# Supplementary material for: Maternal vaccination and protective immunity against Zika virus vertical transmission
Source: Nat Commun. 2019 Dec 12;10:5677. doi: 10.1038/s41467-019-13589-1 (PMC6908683; doi:10.1038/s41467-019-13589-1)
Supplement: Supplementary file 1 — Supplementary Information [file 41467_2019_13589_MOESM1_ESM.pdf]

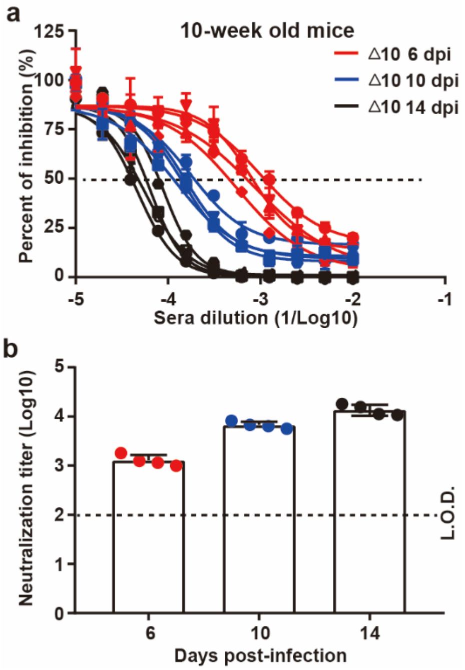

Supplementary Figure 1 Antibody response from non-pregnant A129 female mice after 3′UTR-Δ10-LAV vaccination. Ten-week-old female A129 were subcutaneously immunized with 10^3 FFU 3′UTR-Δ10-LAV. The mice were bled on days 6, 10, and 14 post-infection for measuring neutralizing antibody titers in serum. (**a**) Neutralization curves using an mCherry ZIKV infecting Vero cells. Error bars show the standard deviations from two technical replicates. Dotted line indicates 50% inhibition of viral infection by mouse serum. (**b**) Neutralizing antibody titers on days 6, 10, and 14 post-infection (n = 4 per group). Error bars represent standard deviations. Source data are provided as a Source Data file.

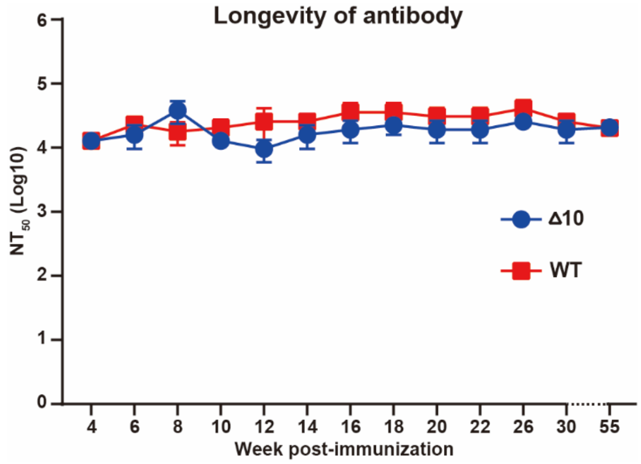

Supplementary Figure 2 Durability of 3'UTR-Δ10-LAV-induced neutralizing antibody activities. Ten-week-old A129 mice were immunized with 10^3 FFU 3′UTR-Δ10-LAV (n = 4) or WT ZIKV PRVABC59 (n = 5). The vaccinated mice were bled biweekly from weeks 4 to 30 and finally at week 55 for neutralizing antibody assays. Error bars represent standard deviations. Source data are provided as a Source Data file.

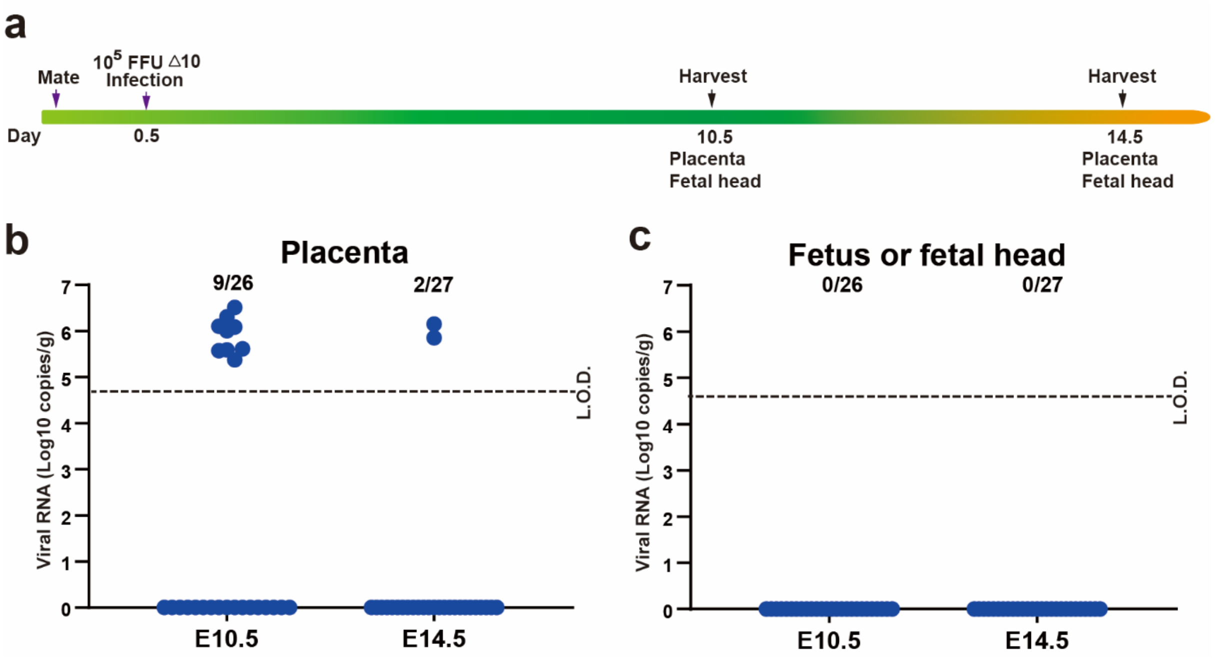

Supplementary Figure 3 The safety of 3′UTR-Δ10-LAV in pregnant A129 mice when vaccinated at E0.5. (**a**) Experimental scheme. Ten- to twelve-week-old pregnant mice were subcutaneously infected with 10^5 FFU WT ZIKV and 3′UTR-Δ10-LAV (Δ10) at E0.5. Placenta and fetus/fetal heads were harvested at E10.5 and E14.5, then samples were analyzed for viral by qPCR. (**b**) Viral RNA load in placenta at E10.5 and E14.5. (**c**) Viral RNA load in fetus/fetal heads at E10.5 and E14.5. Since the fetus was just developed at E10.5, the whole fetus was harvested for analysis. While fetal heads were harvested and used for analysis at E14.5. L.O.D.: limit of detection. Source data are provided as a Source Data file.

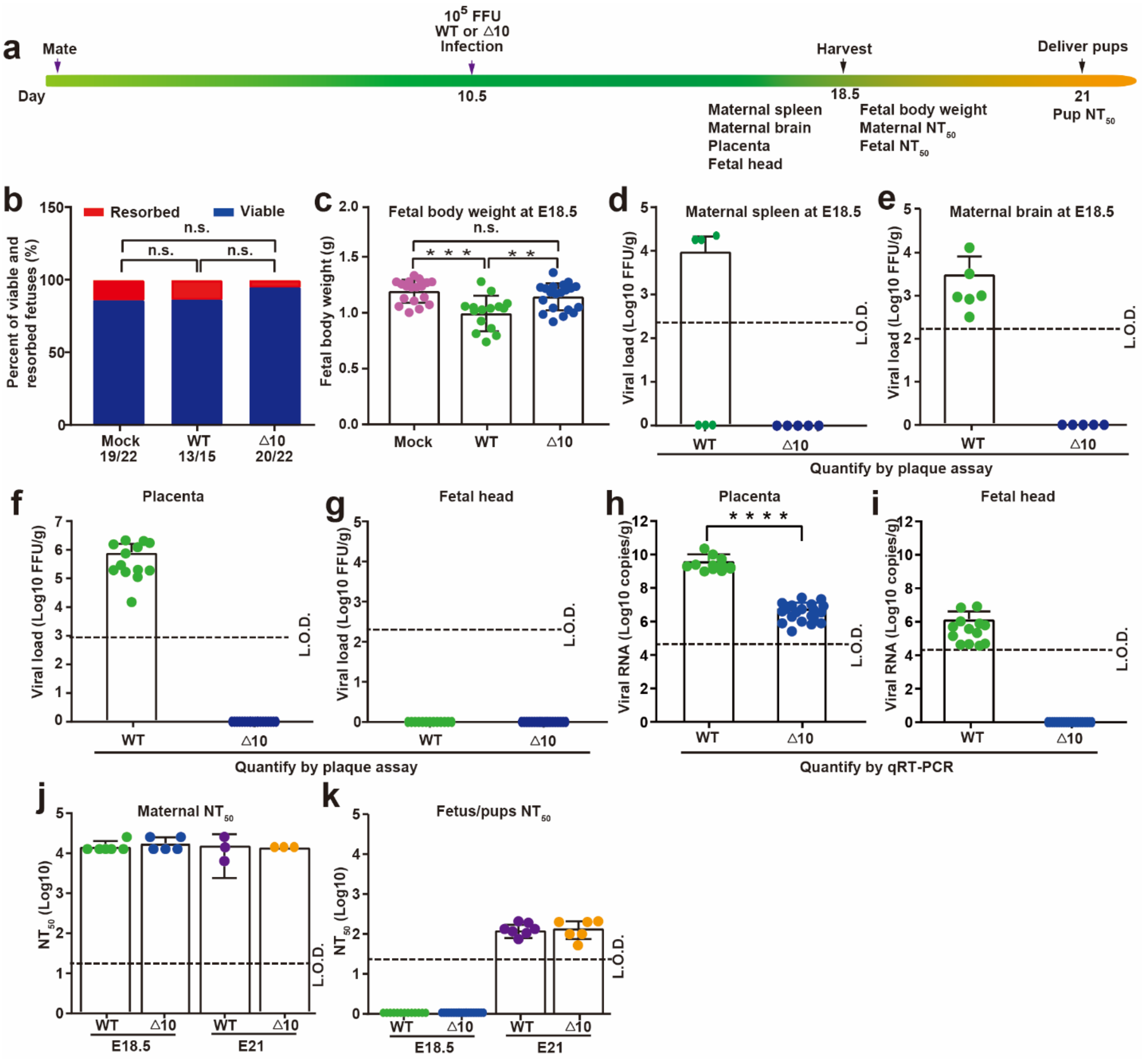

Supplementary Figure 4 The safety of 3′UTR-Δ10-LAV in pregnant A129 mice when vaccinated at E10.5. (**a**) Experimental scheme. Ten- to twelve-week-old pregnant mice were subcutaneously infected with 10^5 FFU WT ZIKV and 3′UTR-Δ10-LAV (Δ10) at E10.5. Maternal and fetal tissues were harvested at E18.5 and analyzed for viral loads and neutralizing antibody titers (NT_{50}). Pups were delivered at E21 and measured for neutralizing antibody titers. (**b**) The percentage of fetuses that were resorbed during pregnancy (n = 22 for placebo; n = 15 for WT ZIKV; n = 22 for Δ10 vaccine; chi-square test [**$p<0.01$]). The numbers of normal fetuses and total fetuses are presented below each group. (**c**) Fetal body weights at E18.5 for placebo, WT ZIKV, and Δ10 groups. Asterisks indicate significant differences (one-way ANOVA). *significant $p<0.5$, non-significant (n.s.) $p>0.5$). Viral loads quantified by plaque assay at E18.5 are presented for maternal spleen (**d**), brain (**e**), placenta (**f**), and fetal head (**g**). Viral RNA quantified by quantitative RT-PCR (qRT-PCR) at E18.5 are presented for placenta (**h**) and fetal head (**i**). Asterisks indicate significant differences (Mann-Whitney test). **** $p<0.0001$. (**j**) Maternal neutralizing antibody titers from WT ZIKV- and Δ10-infected pregnant mice at E18.5 and E21. (**k**) Neutralizing antibody titers in fetuses from WT ZIKV- and Δ10-infected dams at E18.5 and E21. All neutralizing antibody titers

were measured by infecting Vero cells with an mCherry ZIKV. L.O.D.: limit of detection. Error bars represent standard deviations. Source data are provided as a Source Data file.

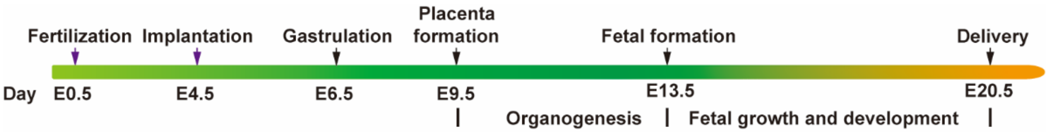

Supplementary Figure 5 Developmental timeline of mouse pregnancy.

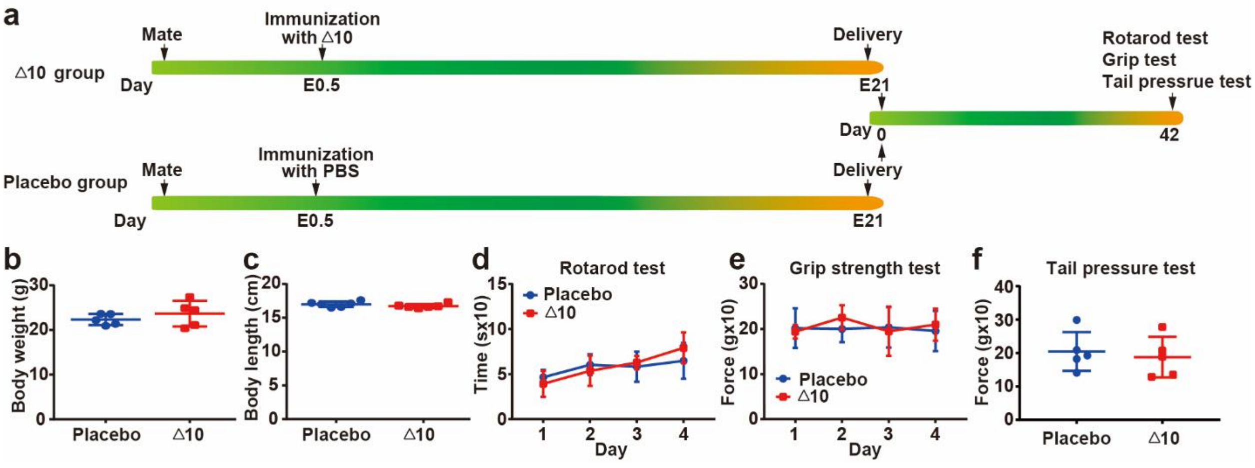

Supplementary Figure 6 The effects of maternal vaccination with 3'UTR-Δ10-LAV on offspring development and behavior. (**a**) Experimental scheme. Ten- to twelve-week-old female A129 mice were mated and immunized with 10^3 FFU 3′UTR-Δ10-LAV or PBS placebo at E0.5. All pregnant mice delivered pups at E21. When the pups reached six weeks of age, their body weights (**b**) and body lengths (**c**) were measured. The pups were then subjected to three different behavior tests: rotarod test (**d**), grip strength test (**e**), and tail pressure test (**f**). As controls, age-matched female mice were injected with PBS and subjected to the same analyses as described for the maternal vaccination group. No significant differences (Mann-Whitney test) were observed in the parameters measured in (**b**-**f**). Error bars represent standard deviations. Source data are provided as a Source Data file.

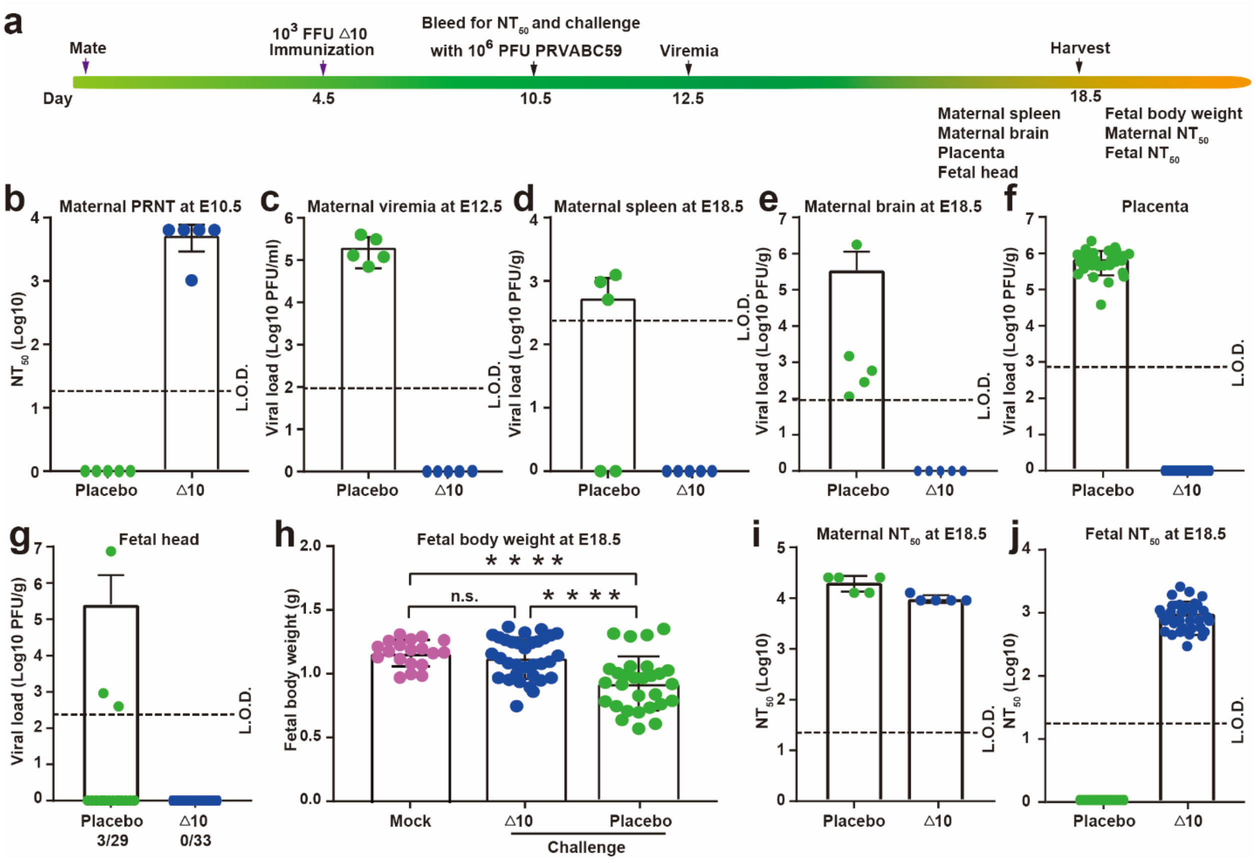

Supplementary Figure 7 The efficacy of 3′UTR-Δ10-LAV in pregnant A129 mice when immunized at E4.5. (**a**) Experimental scheme. Ten- to twelve-week-old pregnant mice were subcutaneously immunized with 10^3 FFU 3′UTR-Δ10-LAV (Δ10) or PBS placebo at E4.5. At E10.5, the pregnant mice were bled for NT_{50} measurement and subcutaneously challenged with 10^6 PFU of ZIKV PRVABC59. Viremia was quantified by at E12.5. Maternal and fetal tissues were harvested for analysis at E18.5. (**b**) Neutralizing antibody titers from PBS- or Δ10-immunized pregnant mice at E10.5. (**c**) Viremia at E12.5. Viral loads at E18.5 are presented for maternal spleen (**d**), brain (**e**), placenta (**f**), and fetal head (**g**). (**h**) Fetal body weights at E18.5 from three experimental groups: no immunization or challenge (mock; *left panel*), Δ10-vaccinated with challenge (*middle panel*), and PBS-immunized with challenge (*right panel*). Asterisks indicate significant differences (one-way ANOVA). ****$p<0.0001$, non-significant (n.s.) $p>0.5$). (**i**) Maternal neutralizing antibody titers from dams at E18.5. (**j**) Fetal neutralizing antibody titers from fetuses at E18.5. Error bars represent standard deviations. Source data are provided as a Source Data file.

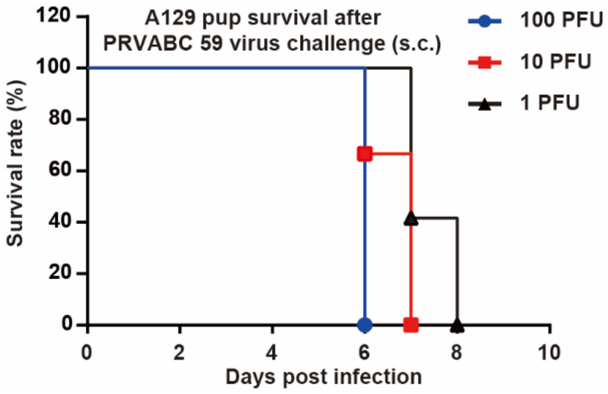

Supplementary Figure 8 Survival curve of A129 neonates after ZIKV PRVABC59 infection. Naïve one-day-old A129 pups were subcutaneously infected with 1, 10, or 100 PFU ZIKV PRVABC59. The infected mice were monitored for survival (n = 12 per group). Source data are provided as a Source Data file.

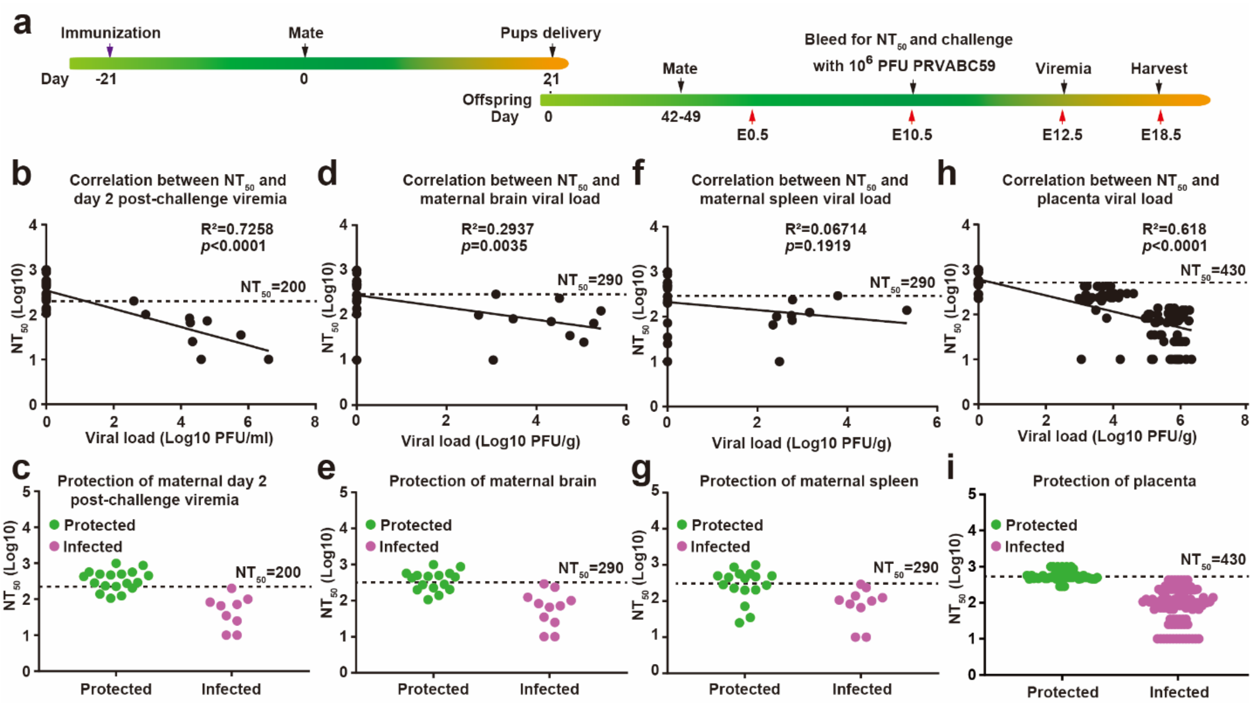

Supplementary figure 9 The minimal neutralizing antibody titers (NT_{50}) required for protection of pregnant A129 mice from apparent ZIKV infection. (**a**) Experimental scheme. Ten- to twelve-week-old female mice were subcutaneously immunized with 10^3 FFU of 3′UTR-Δ10-LAV (Δ10) or PBS on day 21 before pregnancy. The pregnant dams gave birth to pups at full term. The female pups were mated with male mice at six- to seven-week-old. At E10.5, these pregnant mice were bled for neutralizing antibody measurement and subcutaneously challenged with 10^6 FFU ZIKV PRVABC59. The challenged pregnant mice were measured for viremia at E12.5. The maternal and fetal organs were harvested for viral load analysis at E18.5. (**b**) Correlation analysis between NT_{50} titers at E10.5 and viremia at E12.5. (**c**) Comparison of NT_{50} titers and viremia between the protected and infected mice from (**b**). Correlation analyses between organ viral loads (detected at E18.5) and NT_{50} titers (measured at E10.5) are presented for maternal brain (**d**, **e**), spleen (**f**, **g**), and placenta (**h**, **i**). The dotted lines indicate the minimal NT_{50} titers required for protection against viremia and organ infections. Source data are provided as a Source Data file.

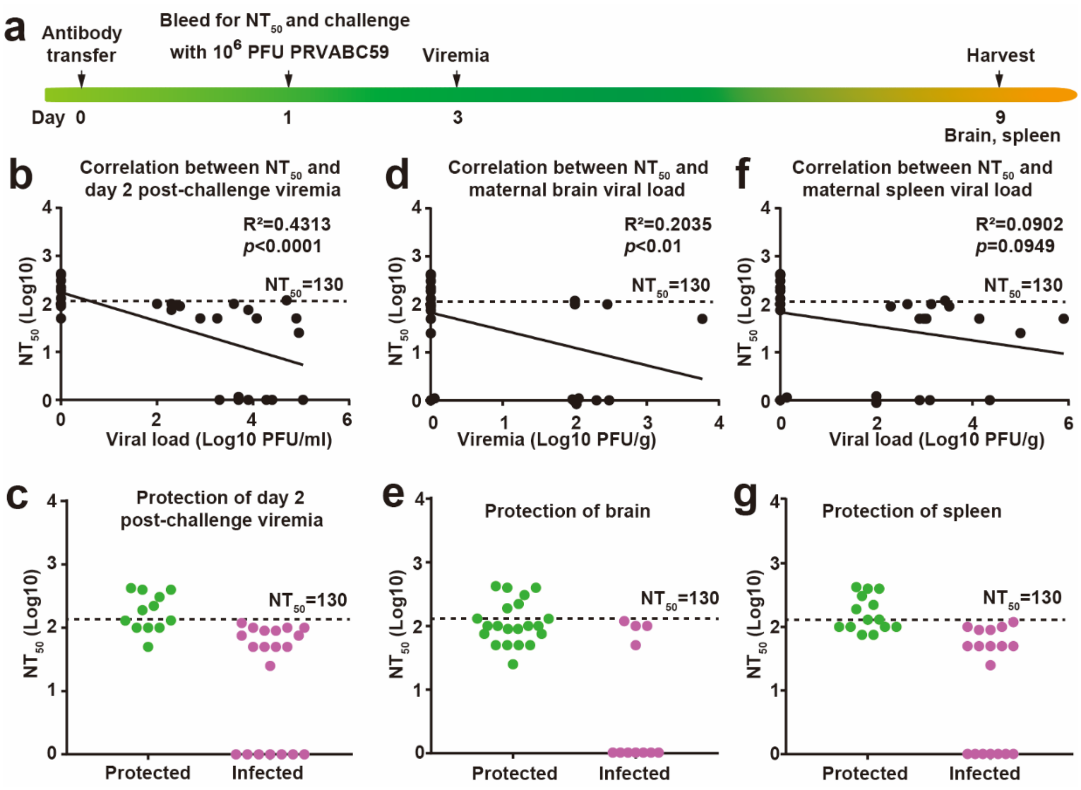

Supplementary Figure 10 The minimal neutralizing antibody titers (NT_{50}) required for protection of non-pregnant A129 mice from apparent ZIKV infection. (**a**) Experimental scheme for passive antibody transfer and efficacy test. Ten- to twelve-week-old female mice were passively transferred with various amounts neutralizing antibodies on day 0. On day 1, the mice were bled for neutralizing antibody measurement and subcutaneously challenged with 10^6 FFU ZIKV-PRVABC59. On day 3, the challenged mice were measured for viremia. On day 9, the infected mice were sacrificed and measured for organ viral loads. (**b**) Correlation analysis between NT_{50} titers on day 1 and viremia on day 3. (**c**) Plot of viremia levels versus different groups of NT_{50} titers from (**b**). Correlation analyses between organ viral loads (detected on day 9) and NT_{50} titers (measured on day 1) are presented for maternal brain (**d**, **e**) and spleen (**f**, **g**). The dotted lines indicate the minimal NT_{50} titers required for protection against viremia and organ infections. Source data are provided as a Source Data file.

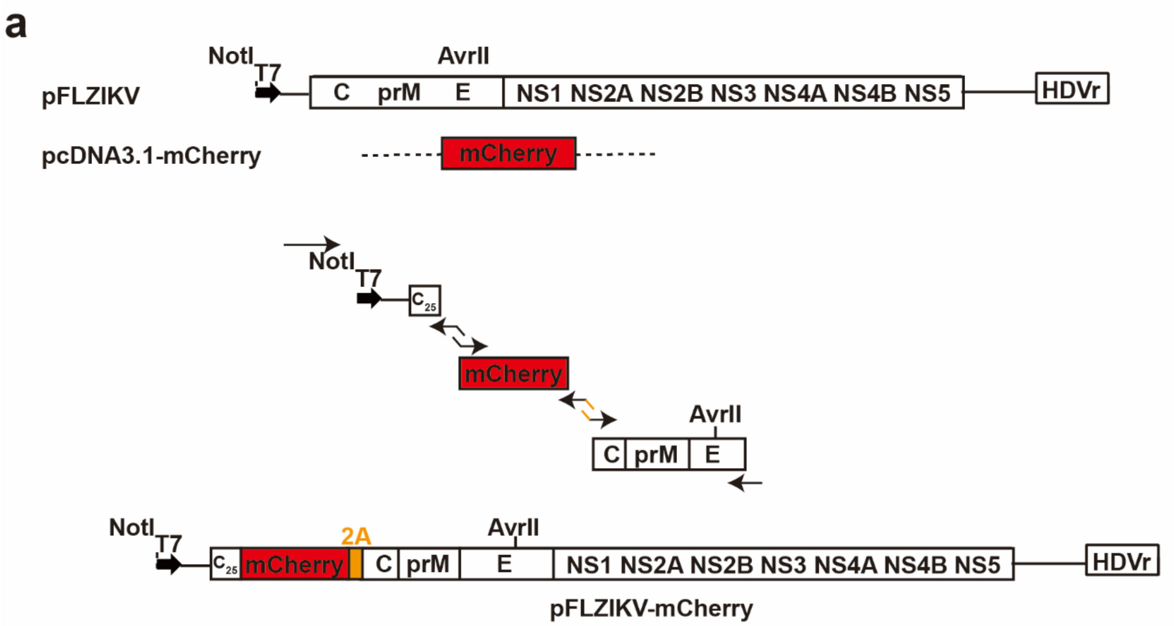

b

Primer name	Sequence (5'-3')
pACYC-14437-F	GCCTACCCGGAACTGAGTGTC
C25-mCherry-F	GTAGCCCGTGTGAGCATGGTGAGCAAGGGCGAG
C25-mCherry-R	GCCCTTGCTCACCATGCTCACACGGGCTACTCC
mCherry-2A-R	GACTCGACGTCTCCCGCCAGCTTGAGAAGGTCAAAATTCAACAGCTGCGTACGC TTGTACAGCTCGTC
2A-Capsid-F	GCTGGCGGGAGACGTCGAGTCCAACCCTGGGCCAATGAAAAACCCAAAGAAGAA
Zika-1818R	TTGCACCATCCATCTCAGCCTCCAGAGCTCCAGC

F, Forward; R, Reverse.

Supplementary Figure 11 Construction of pFLZIKV-mCherry reporter virus. (**a**) Construction of cDNA mCherry reporter ZIKV. To facilitate neutralizing antibody testing, ZIKV-mCherry reporter virus was constructed. Standard PCR procedure was performed to construct pFLZIKV-mCherry. First, a standard overlap PCR was used to create a cassette containing ''NotI-T7 promoter-5'UTR-N-terminal 25 amino acids of capsid protein-mCherry gene-FMDV2A-authentic initiation codon of capsid protein to the unique AvrII site in E protein''. Fragment covering ''NotI to 5'UTR-N-terminal 25 amino acids of capsid protein'' was amplified with the primer pACYC-14437F and C_{25}-mCherry-R using the pFLZIKV as a template. Fragment having "mCherry gene" was amplified from pcDNA3.1-mCherry by primers C_{25}-mCherry-F and mCherry-2A-R. Fragment spanning ''the authentic initiation codon of capsid protein to AvrII unique site in E protein'' (located at nucleotide position 1533 of the viral genome GenBank number KU955593.1)) was amplified with primer 2A-Capsid-F and Zika-1818C using the pFLZIKV as a template. The primer sequences are presented in Table 1. The three fragments were fused together with primers pACYC-14437F and Zika-1818C. Next, the fragment from NotI to AvrII was engineered at the corresponding sites into pFLZIKV, resulting in plasmid pFLZIKV-mCherry. Compared with the wild-type pFLZIKV, pFLZIKV-mCherry contained an extra fragment (representing the first 25 amino acids of C protein-a mCherry gene-FMDV2A) between

the 5’UTR and the complete ORF of the viral genome. All the constructs were verified by DNA sequencing. The rescue of the virus could follow published protocol [1]. (b) Primers used for the construction of cDNA mCherry reporter ZIKV.

Related to Figure 1.

1 Shan, C., Xie, X. & Shi, P. Y. Reverse Genetics of Zika Virus. *Methods Mol Biol* **1602**, 47-58, doi:10.1007/978-1-4939-6964-7_4 (2017).
