# Supplementary material for: Maternal vaccination and protective immunity against Zika virus vertical transmission
Source: Nat Commun. 2019 Dec 12;10:5677. doi: 10.1038/s41467-019-13589-1 (PMC6908683; doi:10.1038/s41467-019-13589-1)
Supplement: Supplementary file 3 — Reporting Summary [file 41467_2019_13589_MOESM3_ESM.pdf]

nature research

Corresponding author(s): Pei-Yong Shi

Last updated by author(s): Nov 5, 2019

Reporting Summary

Nature Research wishes to improve the reproducibility of the work that we publish. This form provides structure for consistency and transparency in reporting. For further information on Nature Research policies, see Authors & Referees and the Editorial Policy Checklist .

Statistics

For all statistical analyses, confirm that the following items are present in the figure legend, table legend, main text, or Methods section.

n/a	Confirmed	
☐	☒	The exact sample size (n) for each experimental group/condition, given as a discrete number and unit of measurement
☐	☒	A statement on whether measurements were taken from distinct samples or whether the same sample was measured repeatedly
☐	☒	The statistical test(s) used AND whether they are one- or two-sided<br>*Only common tests should be described solely by name; describe more complex techniques in the Methods section.*
☐	☒	A description of all covariates tested
☐	☒	A description of any assumptions or corrections, such as tests of normality and adjustment for multiple comparisons
☐	☒	A full description of the statistical parameters including central tendency (e.g. means) or other basic estimates (e.g. regression coefficient) AND variation (e.g. standard deviation) or associated estimates of uncertainty (e.g. confidence intervals)
☐	☒	For null hypothesis testing, the test statistic (e.g. F, t, r) with confidence intervals, effect sizes, degrees of freedom and P value noted<br>*Give P values as exact values whenever suitable.*
☐	☒	For Bayesian analysis, information on the choice of priors and Markov chain Monte Carlo settings
☐	☒	For hierarchical and complex designs, identification of the appropriate level for tests and full reporting of outcomes
☐	☒	Estimates of effect sizes (e.g. Cohen's d, Pearson's r), indicating how they were calculated

Our web collection on statistics for biologists contains articles on many of the points above.

Software and code

Policy information about availability of computer code

Data collection	Neutralizing antibody reading was collected by Cytation 5 Cell Imaging Multi-Mode Reader (Biotek).<br>Flow Flow cytometry data were collected by BD Accuri C6 Flow Cytometer instrument.
Data analysis	Neutralizing antibody was plotted using GraphPad Prism 7 software (La Jolla, CA).<br>Flow Flow cytometry data were analyzed with a Cflow Plus Flow Cytometer (BD Biosciences).<br>All data were analyzed with GraphPad Prism v7.02 software.

For manuscripts utilizing custom algorithms or software that are central to the research but not yet described in published literature, software must be made available to editors/reviewers. We strongly encourage code deposition in a community repository (e.g. GitHub). See the Nature Research guidelines for submitting code & software for further information.

Data

Policy information about availability of data

All manuscripts must include a data availability statement. This statement should provide the following information, where applicable:

- Accession codes, unique identifiers, or web links for publicly available datasets
- A list of figures that have associated raw data
- A description of any restrictions on data availability

It included in the manuscript.

Field-specific reporting

Please select the one below that is the best fit for your research. If you are not sure, read the appropriate sections before making your selection.

Life sciences study design

All studies must disclose on these points even when the disclosure is negative.

Sample size	Sample size was determined to be adequate for statistic analysis and consistency of measurable differences between groups.
Data exclusions	No data exclusions.
Replication	Replicate experiments were successful and reproducible.
Randomization	The mice were randomly allocated to different groups.
Blinding	Investigators were not blinded to groups during experiments. Data reported for mouse experiments are not subjective but rather based on quantitative viral load, neutralizing antibody titer and flow cytometry.

Reporting for specific materials, systems and methods

We require information from authors about some types of materials, experimental systems and methods used in many studies. Here, indicate whether each material, system or method listed is relevant to your study. If you are not sure if a list item applies to your research, read the appropriate section before selecting a response.

Materials & experimental systems

n/a	Involved in the study	
☐	☒	Antibodies
☐	☒	Eukaryotic cell lines
☒	☐	Palaeontology
☐	☒	Animals and other organisms
☒	☐	Human research participants
☒	☐	Clinical data

Methods

n/a	Involved in the study	
☒	☐	ChIP-seq
☐	☒	Flow cytometry
☒	☐	MRI-based neuroimaging

Antibodies

Antibodies used	Describe in the material and method: Viruses, antibodies, and cells section.
Validation	The antibodies were validated by manufacture. House-made antibody was validated by neutralizing test.

Eukaryotic cell lines

Policy information about cell lines

Cell line source(s)	Purchased from American Type Culture Collection (ATCC CCL-81; Bethesda, MD).
Authentication	Authenticated by ATCC.
Mycoplasma contamination	The cell line was tested negative for mycoplasma.
Commonly misidentified lines (See ICLAC register)	None.

Animals and other organisms

Policy information about studies involving animals; ARRIVE guidelines recommended for reporting animal research

Laboratory animals	The information of A129 mice used for experiments can be found in the relevant figure legends and Methods.
Wild animals	The study did not involve wild animal.
Field-collected samples	The study did not involve samples collected from the field.
Ethics oversight	See ethics statement in the Material and Methods.

Note that full information on the approval of the study protocol must also be provided in the manuscript.

Flow Cytometry

Plots

Confirm that:

- [x] The axis labels state the marker and fluorochrome used (e.g. CD4-FITC).
- [x] The axis scales are clearly visible. Include numbers along axes only for bottom left plot of group (a 'group' is an analysis of identical markers).
- [x] All plots are contour plots with outliers or pseudocolor plots.
- [x] A numerical value for number of cells or percentage (with statistics) is provided.

Methodology

Sample preparation	See Material and Methods.
Instrument	BD Accuri C6 Flow Cytometer instrument
Software	Cflow Plus Flow Cytometer (BD Biosciences)
Cell population abundance	See Material and Methods.
Gating strategy	See Material and Methods.

- [x] Tick this box to confirm that a figure exemplifying the gating strategy is provided in the Supplementary Information.
